# Oncological outcomes of breast-conserving surgery versus mastectomy following neoadjuvant chemotherapy in a contemporary multicenter cohort

**DOI:** 10.1038/s41598-025-93491-7

**Published:** 2025-03-16

**Authors:** Francisco Pimentel Cavalcante, Felipe Pereira Zerwes, Ryane Alcantara, Eduardo Camargo Millen, Andre Mattar, Marcelo Antonini, Anne Dominique Nascimento Lima, José Bines, Fabrício Palermo Brenelli, Guilherme Garcia Novita, Anastacio Berretini Junior, Rafael Henrique Szymanski Machado, Alessandra Borba Anton DE SOUZA, Danielle Calheiros Campelo, Rene Aloisio da Costa Vieira, Antônio Luiz Frasson

**Affiliations:** 1 Hospital Geral de Fortaleza, Fortaleza, CE Brazil; 2 Universidade Estadual Paulista Júlio Mesquita Filho (UNESP), Botucatu, SP Brazil; 3 Pontifícia Universidade Católica do Rio Grande do Sul (PUCRS), Porto Alegre, RS Brazil; 4 Americas Oncologia, Rio de Janeiro, RJ Brazil; 5 Hospital da Mulher; and Oncoclínicas, São Paulo, SP Brazil; 6 Hospital do Servidor Público Estadual, São Paulo, SP Brazil; 7 Clínica Lila, Caxias D’Or, Duque de Caxias, RJ Brazil; 8 INCA and São Vicente da Gávea (Rede D’Or), Rio de Janeiro, RJ Brazil; 9 Universidade Estadual de Campinas, Campinas, SP Brazil; 10 Oncoclínicas, São Paulo, SP Brazil; 11 Hospital Salvalus, Hapvida NotreDame Intermédica, São Paulo, SP Brazil; 12 Hospital Federal da Lagoa, Rio de Janeiro, RJ Brazil; 13 Universidade Federal do Ceara (UFC), Fortaleza, CE Brazil; 14 Hospital do Câncer de Muriaé da Fundação Cristiano Varella, Muriaé, MG Brazil; 15 Hospital Israelita Albert Einstein, São Paulo, SP Brazil

**Keywords:** Breast neoplasms, Segmental mastectomy, Mastectomy, Chemotherapy, Neoadjuvant therapy, Locally advanced breast cancer, Cancer, Oncology

## Abstract

To evaluate local recurrence (LR), distant recurrence (DR) and death in non-metastatic patients undergoing breast-conserving surgery (BCS) or mastectomy following current neoadjuvant chemotherapy (NAC) regimens. Patients submitted to NAC in 2013–2023 were evaluated (*n* = 365; mastectomy: 165; BCS: 200). More mastectomy patients were over 70 years old (12.7% versus 7%; *p* = 0.02) and had T4b tumors (16.4% versus 4.5%; *p* = 0.0003), whereas more BCS patients had node-negative axilla (42% versus 31.5%; *p* = 0.02). After a mean follow-up of 65 months (range: 4-124), LR and DR were similar in the mastectomy and BCS groups (4.8% versus 5.0%; *p* = 0.95 and 10.9% versus 9%; *p* = 0.58, respectively). More deaths occurred in the mastectomy group (8.5% versus 3%; *p* = 0.03). Ten-year LR-free survival was higher in the BCS group (98.5% versus 95%; HR: 3.41; 1.09–10.64; *p* = 0.03), while 10-year DR-free survival was similar in both groups (91% BCS versus 89% mastectomy, HR: 1.25; 0.65–2.42; *p* = 0.4). Overall survival was better in the BCS group (97% versus 91.5%; HR: 2.62; 1.06–6.69; *p* = 0.03). Estimated 10-year disease-free survival, stratified according to tumor stage, showed no significant difference except for T4 disease, for which the risk was greater in the mastectomy group (94.5% versus 81.8%; HR: 2.86, 1.54–5.30, *p* = 0.0008). In the multivariate analysis, T3/T4 staging (OR: 4.37, 1.03–21.91; *p* = 0.04) and axillary dissection (OR: 5.11, 1.14–35.52; *p* = 0.04) were associated with LR in the BCS group. In this cohort of patients receiving contemporary NAC, BCS proved to be a safe alternative to mastectomy following treatment with NAC, even in cases of locally advanced BC.

## Introduction

Breast-conserving surgery (BCS) is the preferred surgical treatment for early-stage breast cancer. Similar overall survival (OS) rates have been found with BCS and mastectomy, whereas better quality of life and less mutilation have been shown with BCS^1–6^. More recently, with the advent of organized screening, systemic treatment and a better understanding of the biology of the disease, local recurrence (LR) rates tend to be similar, with several studies concluding that BCS yields even better outcomes compared to mastectomy^7–9^.

Neoadjuvant chemotherapy (NAC) was traditionally used to convert inoperable tumors into operable ones^10^. However, due to its similar efficacy in reducing distant disease and breast cancer-related death compared to adjuvant therapy following surgery, NAC has also been used over the years in patients with initially operable tumors^11–13^. In addition to historically increasing the rate of BCS, particularly in cases that were initially ineligible, numerous other advantages have been identified with NAC, such as allowing response to be evaluated in vivo; cases of residual disease to be selected for additional adjuvant therapies; and axillary surgery and radiotherapy to be de-escalated when a pathologic complete response (pCR) in the axillary lymph nodes is achieved^14–25^.

Increasing the rate of BCS in patients with a good NAC response could, however, also increase LR rates, particularly among patients whose planned mastectomy was converted to BCS^24^. Older studies, in fact, showed an increase in LR in women submitted to BCS following NAC. However, those findings preceded the advent of new drugs that substantially increased pCR in NAC. Achieving pCR represents a good prognosis, particularly in cases of breast cancer with aggressive biology. Moreover, in the adjuvant setting, especially in high-risk disease, modern drugs have had an impact on oncological outcomes, including local control^13–16,24–27^. Other contemporary factors influencing local outcomes include improved understanding of tumor biology, allowing a better selection of cases for NAC; improved imaging methods for evaluating initial cases and response to treatment; and markers to locate the tumor site following NAC^8,28–30^.

The principal objective of the present study was to evaluate the rates of LR, distant recurrence (DR), and death in women undergoing BCS or mastectomy following NAC in a contemporary cohort of patients. Secondary objectives included evaluating disease-free survival (DFS) according to tumor staging, as well as factors related to LR in the two surgical groups.

## Materials and methods

This retrospective multicenter cohort study was conducted with patients treated with BCS or mastectomy following NAC at the Fortaleza General Hospital and the Pontifícia Universidade Católica do Rio Grande do Sul (PUC/RS) between 2013 and 2023. Both hospitals provide healthcare within the Brazilian National Healthcare System (SUS), with specific standards but some limitations insofar as oncological treatment is concerned^31^. Patients with breast cancer cT1-T4, cN0-N3, M0, for whom data on outcomes were available in their medical records, were included in the study. Due to the delay in early detection and initiation of treatment within the SUS, the great majority of patients undergoing NAC are clinical stage III or at least stage II-B. Patients with a prior history of breast cancer, a diagnosis of inflammatory breast cancer, those who failed to complete the proposed NAC regimen, patients who did not undergo surgery, and those without sufficient information on their medical records were excluded from the study. This study was performed in line with the principles of the Declaration of Helsinki. Approval was granted by the Institutional Review Board (IRB), Ethics Committee of Fortaleza General Hospital (CEP-HGF), CAAE number 73596023.4.1001.5040. Informed consent was obtained from all subjects, except from those who could not be contacted and was waived by the CEP-HGF (Approved substantiated document number 7.082.093; date of analysis 08/17/2023).

### Treatment planning, systemic and local therapy

Histopathology and immunohistochemistry results were available for all the patients prior to initiating treatment, with the disease being divided into subtypes: (1) Luminal subtype (hormone receptor-positive/ human epidermal growth factor receptor 2 [HER2]-negative); (2) Luminal HER2 (hormone receptor-positive/HER2-positive); (3) HER2 (hormone receptor-negative/HER-positive); or (4) Triple-negative (absence of hormone receptors and HER2). TNM staging was conducted according to the American Joint Committee on Cancer (AJCC) staging system^32^. Mammography and ultrasonography were routinely used to plan treatment. Magnetic resonance imaging (MRI) of the breast, however, is not universally available within the SUS. Fine needle biopsy or core biopsy of the lymph nodes was performed in cases of suspicious axillary lymph nodes. Systemic staging was initially performed in cases of stage III using a combination of bone scintigraphy, chest radiography and abdominal ultrasound, with computed tomography gradually substituting the latter two over the years. Positron emission tomography was unavailable. In most cases, the lesion was marked before systemic treatment was initiated. Since metallic clips are not generally available within the SUS, the projected area of the tumor was marked using pigmentation (tattooing) on the skin of the breast.

All the patients received NAC regimens containing anthracyclines and taxanes except in the case of HER2 disease when anthracyclines could be excluded and anti-HER2 therapy was administered (TCH – docetaxel, carboplatin, and trastuzumab). Trastuzumab was available for anti-HER2 therapy but pertuzumab was not. Anti-HER2 therapy with trastuzumab was routinely given as adjuvant therapy. Trastuzumab emtansine (T-DM1) was not available for cases of residual HER2 disease, neither were more recent therapies such as pembrolizumab immunotherapy, olaparib for women with the *BRCA* mutation, and adjuvant abemaciclib in hormone-positive tumors. In such cases, tamoxifen or aromatase inhibitors were given, at the discretion of the medical team. The usual recommendation is for five years, since extended endocrine therapy and even premenopausal ovarian suppression are not universally available within the SUS.

Following NAC, new imaging exams were routinely performed to evaluate response to treatment and marking of the tumor/residual area position using guide-wire localization, tumor staining or a metallic clip, if available. BCS or mastectomy was generally performed 5–6 weeks after completion of NAC. If the axilla was clinically negative at the time of surgery, sentinel lymph node biopsy (SLNB) was performed using patent blue dye alone, since radiotracer was unavailable at the time of the study. Over the years, SNLB also began to be used in cases in which the axilla was initially positive but complete clinical and imaging response were achieved. In cases of positive axilla at the time of surgery or when the SLNB was positive, axillary dissection was performed as routine. The margins in all the samples were evaluated, with “no ink on tumor” as the standard for a negative margin at BCS or mastectomy. Positive margins required re-excision with wider margins. Radiotherapy of the whole breast, chest wall and/or lymphatic drainage pathway was performed in accordance with international recommendations, as was postsurgical follow-up.

### Primary and secondary objectives

The primary objectives LR, DR and death were evaluated from the date of surgery in the BCS and mastectomy groups. Likewise, the secondary objectives: LR-free survival (LRFS), DFS - defined as any local/regional/distant recurrence or death, either breast cancer-related or from any cause, and stratified according to tumor staging (cT1-4), DRFS, and OS were also evaluated from the date of surgery. Finally, multivariate logistic regression was performed to evaluate factors associated with LR in the mastectomy and BCS groups. The variables evaluated included age, staging (T3/T4, cN0-3), molecular subtypes (with the HER2 tumors, i.e. luminal HER2 and HER2, being pooled together), axillary dissection, and the use of endocrine therapy.

### Statistical analysis

Clinical and demographic data were described as percentages. Continuous and categorical variables were compared using Student’s t-test and the chi-square test, respectively. Kaplan-Meier curves and the Cox proportional hazard frailty model were constructed for LRFS, DFS (stratified according to T1, T2, T3 and T4 staging), DRFS and OS. Multivariate logistic regression was conducted to evaluate the association between clinical, demographic and treatment-related variables and LR. The program used throughout the entire statistical analysis was GraphPad Prism 8. A significance level of 5% was adopted.

## Results

A total of 365 patients fulfilled the study inclusion criteria. Of these, 165 were submitted to mastectomy and 200 to BCS. The mean age of the patients in the BCS group was 53 years (range 26–81 years) compared to 51 years (range 25–96 years) in the mastectomy group (*p* = 0.328). Women aged ≥ 70 years were more common in the mastectomy group (12.7% versus 7%, *p* = 0.02) and T4b tumors were also more likely in that group (16.4% versus 4.5%; *p* = 0.0003). Clinically negative axilla staging (cN0) was more common in the BCS group (42% versus 31.5%; *p* = 0.02). There were no other statistically significant differences between the groups for any of the other clinical, demographic or treatment-related characteristics (Table 1). After a mean follow-up time of 65 months (range 4-124 months), 18 cases of LR were found: 8 (4.8%) in the mastectomy group and 10 (5.0%) in the BCS group (*p* = 0.95). Furthermore, there was no statistically significant difference in DR: mastectomy 10.9% (18/165) versus BCS 9% (18/200); *p* = 0.58. Conversely, more deaths occurred in the mastectomy group: 8.5% (14/165) versus 3% (6/200); *p* = 0.03 (Table 2).

**Table 1:** Clinical, demographic and treatment-related characteristics of non-metastatic patients who underwent mastectomy or breast-conserving surgery following neoadjuvant chemotherapy.

Characteristics	Mastectomy (*n* = 165)% (n)	BCS (*n* = 200)% (n)	*p*-value
Age (years)			
≤ 40	21.2 (35/165)	21 (42/200)	0.12
41–69	66.1 (109/165)	72 (144/200)	0.97
≥ 70	12.7 (21/165)	7 (14/200)	0.02*
Tumor (T)			
T1	4.8 (8/165)	8 (16/200)	0.17
T2	50.3 (83/165)	63 (126/200)	0.14
T3	26.7 (44/165)	23.5 (47/200	0.66
T4a	1.8 (3/165)	1 (2/200)	0.99
T4b	16.4 (27/165)	4.5 (9/200)	0.0003*
Axillary status			
cN0	31.5 (52/165)	42 (84/200)	0.02*
cN1	50.3 (83/165)	47.5 (95/200)	0.2
cN2	17.6 (29/165)	10.5 (21/200)	0.7
cN3	0.6 (1/165)	0 (0/200)	0.5
Subtypes			
Luminal	45.4 (75/165)	35.5 (71/200)	0.5
Luminal HER2	17.6 (29/165)	26.5 (53/200)	0.1
HER2	18.8 (31/165)	9.5 (19/200)	0.7
Triple-negative	18.2 (30/165)	28.5 (57/200)	0.08
Axillary surgery			
Sentinel node biopsy	44.8 (74/165)	61 (122/200)	0.09
Axillary dissection	55.2 (91/165)	39 (78/200)	0.7
Endocrine therapy			
Yes	64.8 (107/165)	60 (120/200)	0.3
No	35.2 (58/165)	40 (80/200)	
Radiotherapy			
Yes	83 (137/165)	99 (199/200)	0.1
No	17 (28/165)	1 (1/200)	

*BCS* breast-conserving surgery, *p < 0.05, chi-square test.

**Table 2:** Oncological outcomes of non-metastatic patients who underwent mastectomy or breast-conserving surgery following neoadjuvant chemotherapy.

Outcome	Mastectomy (*n* = 165)% (n)	BCS (*n* = 200)% (n)	*p*-value
Local recurrence			
Yes	4.8 (8/165)	5 (10/200)	0.95
No	95.2 (158/165)	95 (191/200)	
Distant recurrence			
Yes	10.9 (18/165)	9 (18/200)	0.58
No	89.1 (147/165)	91 (182/200)	
Death			
Yes	8.5 (14/165)	3 (6/200)	0.03*
No	91.5 (151/165)	97 (194/200)	

*BCS* breast-conserving surgery, *p < 0.05, chi-square test.

**Fig. 1:**
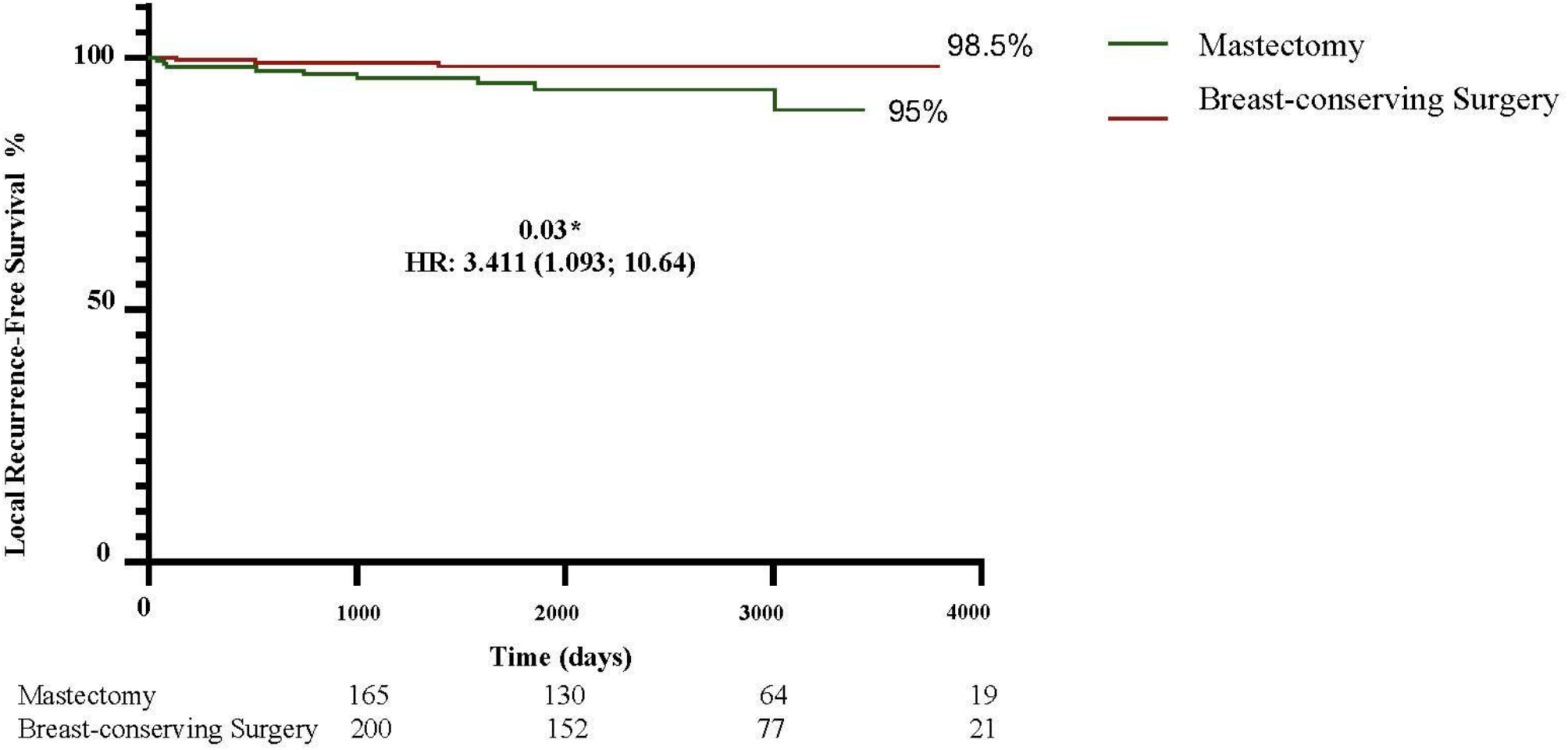
Ten-year local recurrence-free survival rates in the BCS and mastectomy groups.

**Fig. 2:**
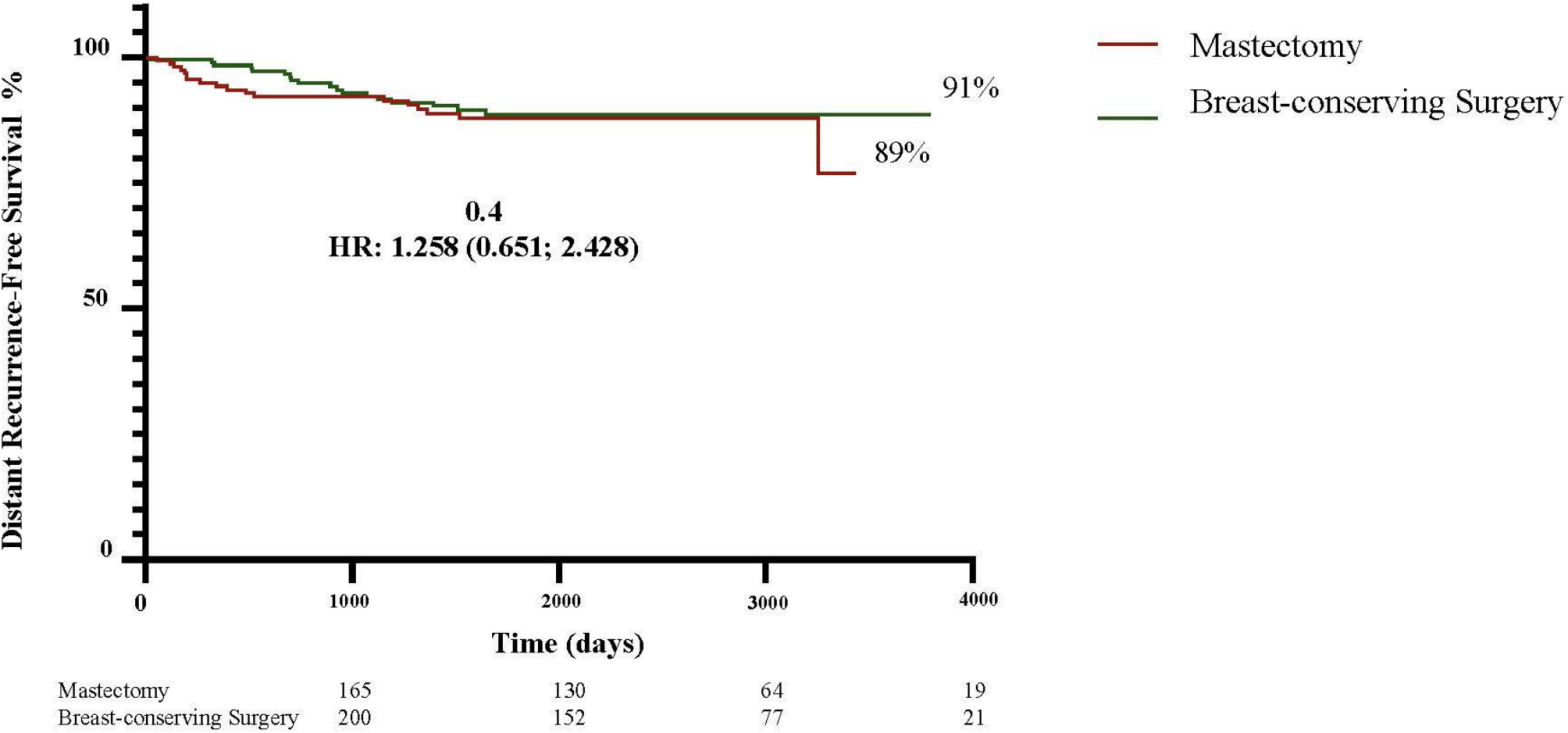
Ten-year distant recurrence-free survival rates in the BCS and mastectomy groups.

**Fig. 3:**
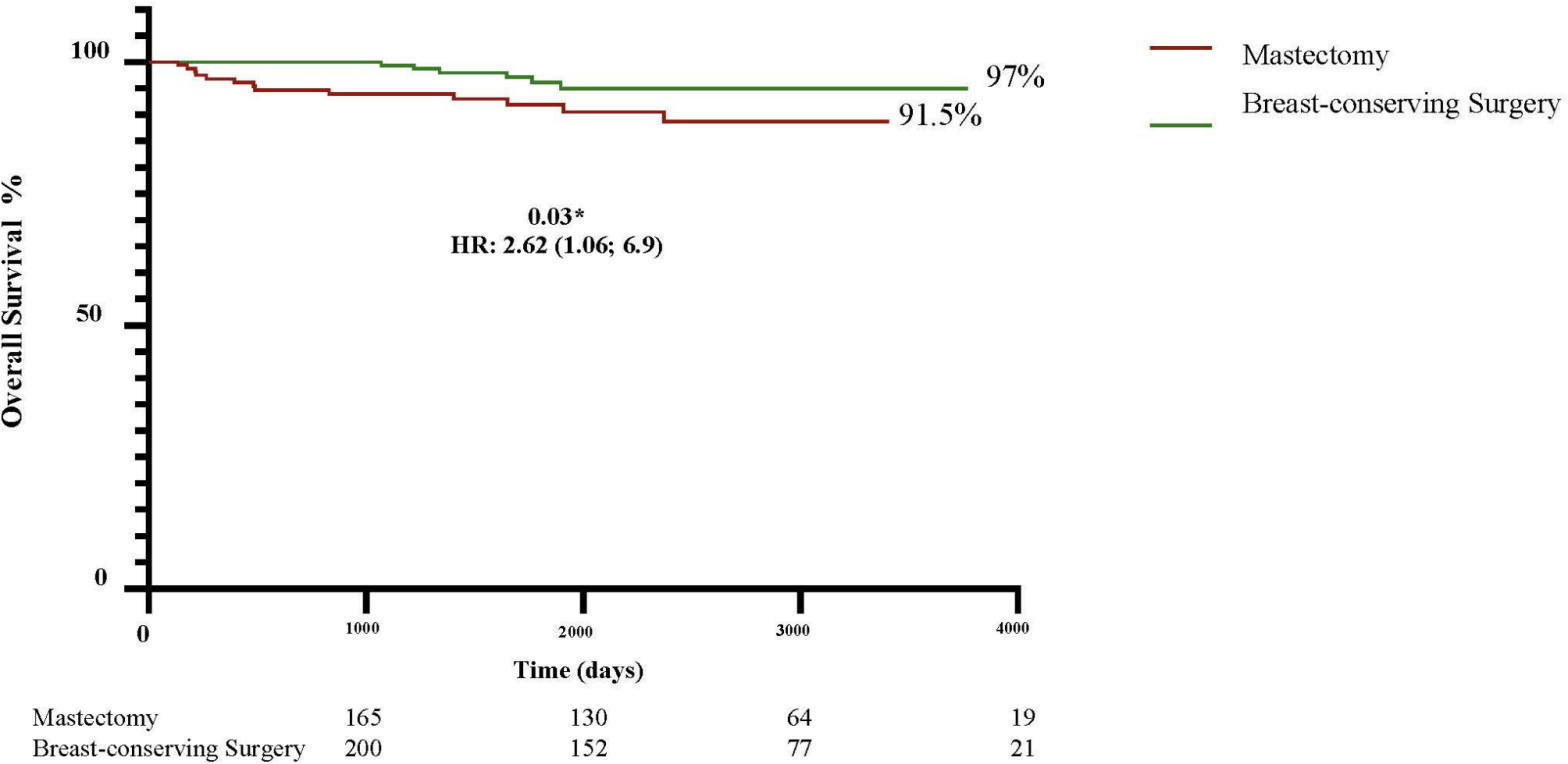
Ten-year overall survival rates in the BCS and mastectomy groups.

**Fig. 4:**
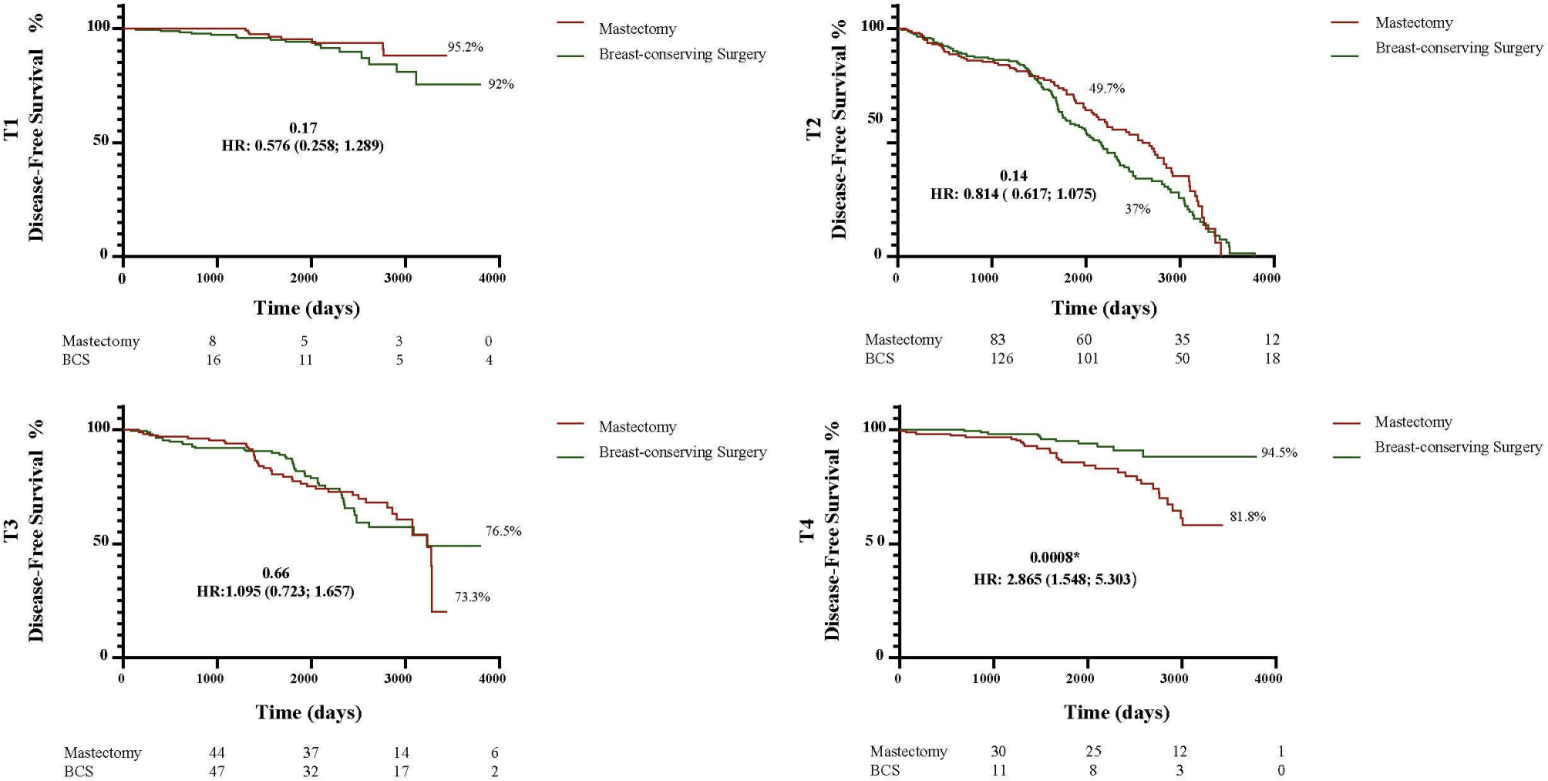
Ten-year disease-free survival rates according to tumor stage in the BCS and mastectomy groups.

The 10-year curves showed a higher LRFS rate in the BCS group compared to the mastectomy group (98.5% versus 95%; HR: 3.41; 1.09–10.64; *p* = 0.03) (Fig. 1). There was no difference in DRFS: 91% in the BCS group compared to 89% in the mastectomy group (HR: 1.258; 0.651–2.428; *p* = 0.04) (Fig. 2). Nevertheless, the estimated OS was higher in the BCS group: 97% versus 91% (HR: 2.62; 1.06–6.49; *p* = 0.03) (Fig. 3). When the DFS was evaluated according to tumor stage, a statistically significant difference was found between the groups only in relation to T4 tumors, with DFS being higher in the BCS group (94.5%) compared to the mastectomy group (81.8%) (HR: 2.865; 1.548–5.303; *p* = 0.0008) (Fig. 4). In the multivariate analysis, stage T3/T4 (OR: 4.37, 1.03–21.91; *p* = 0.04) and axillary dissection (OR: 5.11, 1.14–35.52; *p* = 0.04) were associated with LR in the BCS group (Table 3).

**Table 3:** Multivariate logistic regression of factors associated with local recurrence in non-metastatic patients who underwent mastectomy or breast-conserving surgery following neoadjuvant chemotherapy.

Factors	Mastectomy		BCS	
	Odds ratio (95% CI)	*p*-value	Odds ratio (95% CI)	*p*-value
Age	1.01 (0.96–1.06)	0.52	0.96 (0.90–1.02)	0.31
T3/T4	2.12 (0.50-10.65)	0.31	4.37 (1.03–21.91)	0.04*
cN0	0.28 (0.01–1.63)	0.24	1.37 (0.31–5.95)	0.66
cN1	0.98 (0.22–4.30)	0.98	0.65 (0.13–2.73)	0.56
cN2	2.88 (0.56–12.50)	0.16	1.30 (0.06–7.88)	0.81
Luminal	2.07 (0.49–10.38)	0.33	0.60 (0.08–2.72)	0.55
HER2	0.23 (0.01–1.38)	0.18	0.54 (0.07–2.43)	0.46
Triple-negative	1.00 (0.13–4.94)	> 0.99	2.62 (0.60-11.45)	0.18
Axillary dissection	6.08 (1.04–115.10)	0.09	5.11 (1.14–35.52)	0.04*
Endocrine therapy	0.89 (0.21–4.51)	0.88	0.36 (0.07–1.54)	0.18

*BCS* breast-conserving surgery, * *p* < 0.05, multinomial logistic regression.

## Discussion

This contemporary study demonstrated the comparability between BCS and mastectomy following NAC so far as oncological outcome is concerned, particularly the LR rate of around 5% irrespective of the initial tumor stage, even in locally advanced breast cancer (LABC) (T3). This analysis gives further support to the concept that BCS may constitute a surgical option for breast cancer patients following NAC. Furthermore, these results may reflect the impact of modern management of early-stage breast cancer, including the improvement in pharmacological treatment, advances in knowledge on the biology of the disease, and adequate planning before and after NAC, particularly the use of pre-treatment markers of the initial disease, and even possibly in cases of patients not initially eligible for BCS^6–9^.

These data differ from earlier reports that showed a higher rate of LR with BCS. A meta-analysis of 10 randomized studies conducted between 1983 and 2002 evaluated LR and mortality in 4,756 patients with early-stage breast cancer who underwent surgery either after NAC or prior to adjuvant chemotherapy, using the same regimen of drugs^24^. BCS was higher in patients treated with NAC compared to those submitted to primary surgery (65% versus 49%). After a mean follow-up time of nine years, LR was higher with NAC compared to upfront surgery, with an estimated 15-year LR of 21.4% compared to 15.9%, an absolute increase of 5.5% (95%CI: 2.4–8.6) and a rate ratio of 1.37 (95%CI: 1.17–1.61; *p* = 0.0001). Nevertheless, LR was not associated with any significant increase in DR (38.2% for NAC versus 38%) or breast cancer-related mortality (34.4% for NAC versus 33.7%). On the other hand, more recent studies have shown low rates of LR in BCS following NAC^33,34^, corroborating those found in the present study. An analysis of cT1-3 patients treated between 2013 and 2018 compared patients who underwent BCS and were already eligible prior to NAC with others who were initially BCS-ineligible but became eligible following NAC (*n* = 282; 41%) and with women who were BCS-ineligible, became eligible after NAC, but chose to undergo mastectomy (*n* = 160; 23%)^33^. After a mean follow-up time of 35 months, LR was found in 22 patients (3.2%), with no difference between the groups (*p* = 0.17).

Many factors may explain the higher rate of LR in older studies, including the systemic treatment used at that time, as the new treatment regimens have improved local control, either with NAC or adjuvant treatment^7,27^. The great majority of the patients included in those older studies did not have access to drugs such as anthracyclines and taxanes in the same neoadjuvant regimen, unlike the current approach in which most women undergoing NAC use these regimens, including those in the present study. This difference could have had an impact on outcome. Studies have shown that adding taxanes to the anthracyclines not only increases the pCR rate but also reduces LR^27^. Another relevant factor is anti-HER therapy, which was unavailable at that time. The addition of trastuzumab impacted the clinical outcome of patients with HER2 overexpression, including an increase in pCR and a decrease in LR and DR^7^. More recently, the use of dual HER2 blockade with trastuzumab plus pertuzumab in NAC for HER2 + breast cancer also had a significant effect on pCR^35^.

The use of NAC has increased in recent years, particularly with the aim of achieving pCR. The addition of new drugs that increased the pCR rate had an effect on the rates of BCS, making NAC preferable in many cases. In TN tumors, the addition of platinum-based NAC increased the pCR rate to over 50%, accompanied by an increase in BCS eligibility^25,26^. Recently, the use of pembrolizumab further increased the pCR in TN tumors (> 60%) to a level closer to that found in HER2 disease^36,37^. Increasing pCR rates may also reduce the need for radiotherapy. The NSABP-51 study showed that omitting radiotherapy of the chest wall and lymphatic drainage pathway in mastectomy and BCS, respectively, in patients with initially positive axilla (cN1) and who achieved pCR in the lymph nodes following NAC, had no effect on oncological outcome^23^. On the other hand, failure to achieve pCR may help select cases for the use of adjuvant therapies that can also play a relevant role in locoregional control, characterizing a strategic use of NAC in high-risk disease. This applies not only when pCR was not achieved but also in the case of adverse subtypes, with the inclusion of additional adjuvant therapies such as T-DM1 in HER2 disease, capecitabine in triple-negative tumors, olaparib in tumors with the *BRCA* mutation and abemaciclib in hormone-positive tumors, with a consequent effect on oncological outcomes^14–17^. In the present study, many of these treatments were not yet available due to access issues within the SUS. Anti-HER2 therapy with trastuzumab is available, but T-DM1 and pertuzumab are not. Furthermore, pembrolizumab, olaparib and abemaciclib are also unavailable, leading us to presume that the oncological safety of surgery could increase should they become available.

The increased response to treatment introduced a new challenge in surgical planning, particularly in BCS, with the inclusion of imaging prior to and after NAC being crucial in rendering surgery more precise. This factor could have affected the present results. The quality of mammograms has increased greatly over the years and, furthermore, the addition of new imaging methods such as ultrasonography and MRI of the breast has resulted in better local staging of the disease compared to mammography alone in patients undergoing NAC. These techniques enable residual disease to be identified, allowing the lesion to be located prior to surgery or permitting the detection of metallic clips if the lesion is no longer visible in the image. In general, MRI of the breast is not available here; however, all the patients in this study underwent mammograms and ultrasonography of the breast prior to and following systemic treatment, with localization of the residual lesion when evident or of clipping, whenever available, if the lesion could no longer be identified. In such cases, localization of the tumor bed is crucial in assuring the safety of surgery. One study evaluated the role of clipping in BCS following NAC in 145 clipped patients compared to 228 patients who underwent BCS without clipping^29^. After a mean follow-up of 49 months, the estimated 5-year LR rate was 98.6% for the cases in which tumors were previously marked with a clip versus 91.7% (*p* = 0.02). In a multivariate analysis, omission of the clip in the tumor bed was associated with a hazard ratio of 3.69 for an increase in LR. Unfortunately, metallic clips are not yet universally available within the SUS. In most cases, skin tattooing was performed on the tumor projection. This strategy, despite being feasible and facilitating the possible projection of the initial lesion, may not be as precise as marking the tumor site with a clip, and could lead to the unnecessary resection of a greater volume of tissue. A retrospective analysis compared localization of the tumor in patients who underwent NAC and BCS between 1999 and 2009 with the use of clips (*n* = 31) or skin tattooing (*n* = 118). The volume of tissue resected was significantly greater in the group with skin tattoos (268 cm^3^ versus 143 cm^3^; *p* < 0.04)^30^.

In the present study, around 30% of cases with initial stage T3/T4 underwent BCS following NAC. This was associated with a greater rate of LR in the multivariate analysis; however, regarding stage-specific DFS (T3/T4), there were no statistically significant differences compared to mastectomy. Indeed, BCS in locally advanced breast cancer (LABC), although a subject of debate, has gained in popularity over recent years following studies reporting its safety^38–40^. A meta-analysis with 16 studies involving patients with LABC compared BCS (*n* = 1,465) with mastectomy (*n* = 2,066) following good response to NAC^40^. Most of those older studies did not use anthracyclines or taxanes in the same neoadjuvant regimen. Following a mean follow-up time of > 27 to 76.8 months, there was no statistically significant difference in LR (OR = 0.83; 0.60–1.15; *p* = 0.26); however, DFS was higher with BCS compared to mastectomy (OR = 2.35, 1.84–3.01), as was OS (OR = 2.12; 1.51–2.98; *p* < 0.01). Those results corroborate the present analysis. Another important factor is that with the increased response to NAC in HER2 and triple-negative tumors and the associated high rates of pCR, BCS in LABC appears to represent a viable option^41,42^. In the present study, around 65% of tumors had these characteristics.

There are some limitations associated with this study. First, it is a retrospective study and selection biases could have affected the results. Indeed, there are some differences between the groups with respect to their clinical and demographic characteristics. The difference in results observed in T4 tumors, for example, could be explained by the small number of patients. Furthermore, pCR data are not available in our study, although it is not a mandatory condition for BCS^43^. Limited access to targeted therapies (pertuzumab, T-DM1, pembrolizumab, olaparib, and abemaciclib) is also another limitation. Oncological outcomes in patients treated in institutions that have access to these new technologies may be different. The volume of oncoplastic surgeries used, the use of cavity shaving and margin width, are also not available, factors that could affect regional control. In the present study, the concept “no ink on tumor” was considered the ideal margin following NAC; however, the appropriate margin in such cases has been the subject of debate. The pattern of response to chemotherapy can impact margins and consequently oncological outcomes^44^. Nevertheless, some retrospective studies have shown no association between margin width and poorer oncological outcomes (LR, DFS or OS), suggesting that “no ink on tumor” could be adequate in BCS following NAC^45,46^. BCS improve Quality-of-Life (QoL) and functional outcomes in the long term. A recent study compared long-term QOL outcomes, mean diagnostic interval of 9 years, in patients undergoing BCS (*n* = 631) compared to mastectomy with reconstruction (*n* = 584): the latter was associated with worse long-term sexual well-being compared to BCS^47^. We do not have data on patient-reported outcome measures (PROMs) reported, which is another limitation of our study. On the other hand, this is a multicenter, contemporary study, with a mean follow-up time of over five years, conducted in institutions with limited financial resources; hence, without the benefit of various technologies that could have had a positive effect on oncological outcome, including local control.

## Conclusion

In this contemporary, multicenter, retrospective cohort of patients treated for non-metastatic breast cancer in institutions with limited financial resources, BCS proved to be a safe option to mastectomy following treatment with NAC, even in cases of LABC. These findings corroborate other recent analyses. Further studies are required.

## Data Availability

The datasets generated and/or analyzed during the current study are available at Open Science Framework (OSF) (https://osf.io/uv5rg/?view_only=f5ab356ecc524b09a57c01a20c13090d) or directly from the corresponding author upon reasonable request.

## Abbreviations

AJCC: American Joint Committee on Cancer
BCS: Breast-conserving surgery
DFS: Disease-free survival
DR: Distant recurrence
LABC: Locally advanced breast cancer
LR: Local recurrence
LRFS: Local recurrence-free survival
MRI: Magnetic resonance imaging
NAC: Neoadjuvant chemotherapy
OS: Overall survival
pCR: Pathologic complete response
SLNB: Sentinel lymph node biopsy
SUS: Brazilian National Healthcare System
T-DM1: Trastuzumab emtansine
